# Supplementary material for: Evolving or immutable - phase I solid tumor trials in the era of precision oncology
Source: Invest New Drugs. 2024 May 22;42(3):326–34. doi: 10.1007/s10637-024-01445-z (PMC11164775; doi:10.1007/s10637-024-01445-z)
Supplement: Supplementary file 1 — Supplementary Material 1 [file 10637_2024_1445_MOESM1_ESM.docx]

**Supplementary Material**

**Search Strategy**

The search strategy was composed of the following combination of keywords and database-specific subject headings, including the following search terms and variations of each term. Search results were imported into EndNote 20 for removal of duplicate citations.

**PubMed (NCBI) Search Strategy**

**Date Last Searched: 3/9/2022**

**Results: 2,830**

**Limits: Publication dates 1/1/2000 – 12/31/2020, Select journals (Annals of Oncology, British Journal of Cancer, Cancer Discovery, Clinical Cancer Research, Investigational New Drugs, JAMA Oncology, Journal of Clinical Oncology, Lancet, Lancet Oncology, Molecular Cancer Therapeutics, The New England Journal of Medicine, The Oncologist)**

("Carcinoid Tumor"[Mesh] OR "Carcinoma"[Mesh] OR "Glioma"[Mesh] OR "Melanoma"[Mesh] OR "Meningioma"[Mesh] OR "Neoplasms, Germ Cell and Embryonal"[Mesh] OR "Neurilemmoma"[Mesh] OR "Sarcoma"[Mesh] OR adenocarcinoma*[tiab] OR adeno-carcinoma*[tiab] OR adenocancer[tiab] OR adenocancers[tiab] OR adeno cancer[tiab] OR adeno cancers[tiab] OR argentaffinoma[tiab] OR argentaffinomas[tiab] OR blastoma[tiab] OR blastomas[tiab] OR carcinoid[tiab] OR carcinoids[tiab] OR carcinoma*[tiab] OR carcinosarcoma[tiab] OR carcinosarcomas[tiab] OR carcinosis[tiab] OR epithelial cancer*[tiab] OR epithelial malignan*[tiab] OR ganglioglioma[tiab] OR germ cell cancer*[tiab] OR germ cell malignan*[tiab] OR germ cell neoplas*[tiab] OR germ cell tumor*[tiab] OR germ cell tumour*[tiab] OR germinal cancer*[tiab] OR germinal malignan*[tiab] OR germinal neoplas*[tiab] OR germinal tumor*[tiab] OR germinal tumour*[tiab] OR germinal cell cancer*[tiab] OR germinal cell neoplas*[tiab] OR germinal cell tumor*[tiab] OR germinal cell tumour*[tiab] OR glia cancer*[tiab] OR glia tumor*[tiab] OR glial cell neoplas*[tiab] OR glial cell tumor*[tiab] OR glial cell tumour*[tiab] OR germinoma[tiab] OR germinomas[tiab] OR glioblastoma[tiab] OR glioblastomas[tiab] OR glioma[tiab] OR gliomas[tiab] OR histiosarcoma[tiab] OR malignant epithelial neoplas*[tiab] OR malignant epithelial tumor*[tiab] OR malignant epithelial tumour*[tiab] OR malignant meningeal neoplas*[tiab] OR malignant meningeal tumor*[tiab] OR malignant meningeal tumour*[tiab] OR malignant meningioma[tiab] OR malignant meningiomas[tiab] OR malignant neurilemmoma[tiab] OR malignant neurilemmomas[tiab] OR malignant neurilemoma[tiab] OR malignant neurilemomas[tiab] OR malignant neurolemmoma[tiab] OR malignant schwann cell[tiab] OR malignant schwannoma[tiab] OR melanocarcinoma*[tiab] OR melanoma[tiab] OR melanomas[tiab] OR melanomalignoma[tiab] OR melanomatosis[tiab] OR meningeal cancer*[tiab] OR meningeal malignan*[tiab] OR meningeal metastas*[tiab] OR meningeal metastatic[tiab] OR meninges cancer*[tiab] OR meningioma malignan*[tiab] OR meningiomas malignan*[tiab] OR mesenchymal cancer*[tiab] OR mesenchymal malignan*[tiab] OR mesenchymal metastatic[tiab] OR mesenchymal neoplas*[tiab] OR mesenchymal tumor*[tiab] OR mesenchymal tumour*[tiab] OR microcarcinoma*[tiab] OR micro-carcinoma*[tiab] OR neurilemoma malignan*[tiab] OR neurilemmoma malignan*[tiab] OR neuroepithelial cancer*[tiab] OR neuroepithelial tumor*[tiab] OR neuroepithelial tumour*[tiab] OR neuro-epithelial tumor*[tiab] OR neuro-epithelial tumour*[tiab] OR neuroepithelioma[tiab] OR neuroepitheliomas[tiab] OR neuro-epithelioma[tiab] OR neurosarcoma[tiab] OR neurosarcomas[tiab] OR neuro-sarcoma[tiab] OR nevocarcinoma[tiab] OR nevocarcinomas[tiab] OR nevo-carcinoma[tiab] OR nevo-carcinomas[tiab] OR naevocarcinoma[tiab] OR naevocarcinomas[tiab] OR naevo-carcinoma[tiab] OR non-epithelial cancer*[tiab] OR non-epithelial malignan*[tiab] OR non-epithelial neoplas*[tiab] OR non-epithelial tumor*[tiab] OR non-epithelial tumour*[tiab] OR sarcoma[tiab] OR sarcomas[tiab] OR schwannoma malignan*[tiab] OR schwannomas malignan*[tiab] OR (("Neoplasms"[Mesh] OR cancer*[tiab] OR malignan*[tiab] OR metastas*[tiab] OR metastatic[tiab] OR neoplas*[tiab] OR oncology[tiab] OR oncologies[tiab] OR oncologic*[tiab] OR tumor*[tiab] OR tumour*[tiab]) AND solid[tiab])) AND ((("Clinical Study" [Publication Type:NoExp] OR "Clinical Trial"[Publication Type:NoExp] OR "Controlled Clinical Trial"[Publication Type] OR clinical study[tiab] OR clinical studies[tiab] OR clinical trial[tiab] OR clinical trials[tiab] OR control trial[tiab] OR control trials[tiab] OR controlled trial[tiab] OR controlled trials[tiab] OR drug evaluation[tiab] OR drug evaluations[tiab] OR drug trial[tiab] OR drug trials[tiab] OR evaluation study[tiab] OR evaluation studies[tiab] OR randomised study[tiab] OR randomised studies[tiab] OR randomised trial[tiab] OR randomised trials[tiab] OR randomized study[tiab] OR randomized studies[tiab] OR randomized trial[tiab] OR randomized trials[tiab] OR trial[tiab] OR trials[tiab]) AND (phase I[tiab] OR phase Ia[tiab] OR phase Ib[tiab] OR phase 1[tiab] OR phase 1a[tiab] OR phase 1b[tiab] OR phase Ia/Ib[tiab] OR phase Ia-Ib[tiab])) OR "Clinical Trial, Phase I"[Publication Type] OR phase I trial[tiab] OR phase I trials[tiab] OR phase Ia trial[tiab] OR phase Ib trial[tiab] OR phase Ib trials[tiab] OR phase 1 trial[tiab] OR phase 1 trials[tiab] OR phase 1a trial[tiab] OR phase 1b trial[tiab] OR phase 1b trials[tiab] OR phase I study[tiab] OR phase I studies[tiab] OR phase Ia study[tiab] OR phase Ib study[tiab] OR phase Ib studies[tiab] OR phase 1 study[tiab] OR phase 1 studies[tiab] OR phase 1a study[tiab] OR phase 1b study[tiab] OR phase 1b studies[tiab] OR phase Ia/Ib trial[tiab] OR phase Ia-Ib trial[tiab] OR phase Ia/Ib study[tiab] OR phase Ia-Ib study[tiab] OR FDA phase I[tiab] OR FDA phase 1[tiab]) AND (2000/1/1:2020/12/31[pdat]) AND ("Annals of oncology : official journal of the European Society for Medical Oncology"[Journal] OR "British journal of cancer"[Journal] OR "Cancer discovery"[Journal] OR "Clinical cancer research : an official journal of the American Association for Cancer Research"[Journal] OR "Investigational new drugs"[Journal] OR "JAMA oncology"[Journal] OR "Journal of clinical oncology : official journal of the American Society of Clinical Oncology"[Journal] OR "Lancet (London, England)"[Journal] OR "The Lancet. Oncology"[Journal] OR "Molecular cancer therapeutics"[Journal] OR "The New England journal of medicine"[Journal] OR "The oncologist"[Journal])

**Statistical Methods**

Categorical variables were summarized as frequencies and as percents of non-missing values.  Logistic regression was used to estimate odds ratio’s of single and multivariable associations of selected predictor variables on the probability of phase II investigation and regulatory approval.  Forest plots of variable effects as odds ratios with 90%, 95%, and 99% confidence intervals were constructed.  Analyses were conducted using the R package version 4.3.0.

**Exploratory Analysis**

The trial features assessed to explore their association with whether the study agent was further studied in a phase II trial and eventually obtained regulatory licensure for the indication investigated in phase I testing included the following: first-in-human testing, funding for the clinical trial, center(s) where the study was conducted, study recruitment location(s), drug class of the study agent, EC use and type of DES.

**Definitions**

A new drug in the context of a drug combination was identified as new if it did not possess prior regulatory licensure in any disease space. A drug was categorized as established if it possessed regulatory licensure in any disease setting prior to testing in the study. A drug’s mechanism of action was defined as immunotherapy if the drug primarily functioned by engaging with the immune system, (e.g., monoclonal antibodies directed against an immune checkpoint or an immune target, or an anti-cancer vaccine). A study drug’s mechanism of action was defined as a DNA damage repair inhibitor if it was directed against a target involved in DNA damage repair pathways (e.g. PARP, ATM, ATR). A drug’s mechanism of action was defined as a targeted agent if the drug had a specific biologic target (e.g. proteomic targets like EGFR, VEGF or HER2) but was not a DNA damage repair inhibitor or an immunotherapeutic agent. A study drug’s mechanism of action was classified as “other” if it did not meet to criteria to be considered chemotherapy, immunotherapy, a DNA damage repair inhibitor, or a targeted agent. A study was categorized as requiring genomic or proteomic inclusion criteria if a genomic or proteomic marker was mandated for study enrollment. Biopsies were classified as mandatory if they were clearly specified in the methods section for a subset of the study population or entire study population. A grade 5 adverse event (AE) was recorded if it was designated as treatment related. If a designation about the etiology of a grade 5 AE was not recorded, it was assumed to be treatment related and recorded. The “other” designation in the study sponsorship category was used if a study had multiple sponsors or other means of sponsorship (e.g. academic institutions, grants). The “other” tumor type category included tumors other than: prostate, breast, lung, colorectal, gynecologic (e.g. ovarian, cervical, endometrial, vulvar), sarcoma, neuroendocrine, head and neck, melanoma, renal cell carcinoma, bladder, esophageal, pancreatic, hepatocellular and gastric cancer. We only classified cohorts as “expansion cohorts” if they were independently reported and clearly defined as distinct entities from the escalation cohort. Treatment emergent adverse events (TEAEs) were designated as any AE regardless of relationship to the investigational agent. Treatment related AEs (TRAEs) were designated as any AE attributed to the investigational agent. A study agent(s) was noted as being tested subsequently in a phase II trial if the drug was used in a phase II trial in the same way it was tested and/or combined in the phase I study. A study agent(s) was noted as leading to regulatory approval if it was granted licensure based upon how it was tested and/or combined in the phase I study.

**Supplementary Tables and Figures**


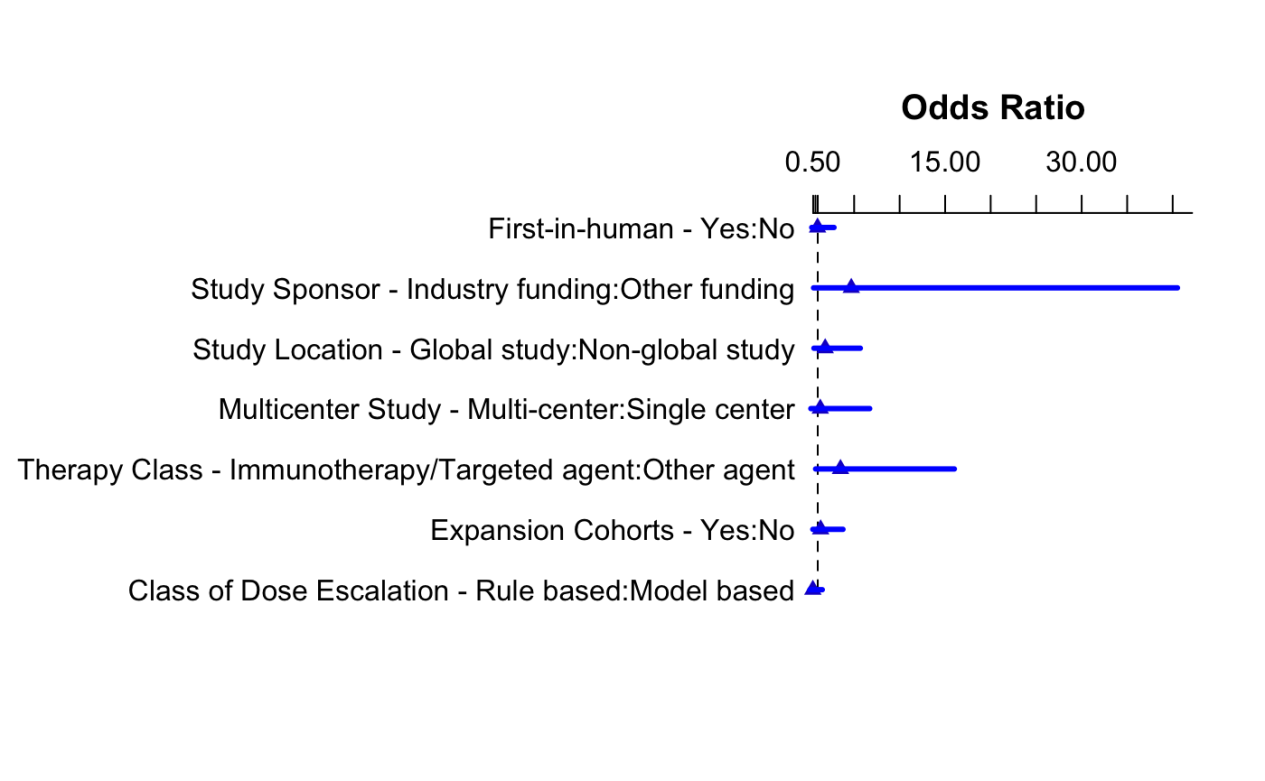


**Supplementary Figure 1: Multi-variate analysis of factors associated with subsequent regulatory approval**

**Supplementary Table 1: Dose-Limiting Toxicities and Grade 3/4 Adverse Events**

| **Most Common Grade ¾ AE’s(N=421)** | |
| --- | --- |
| Hematologic | 156 (37.1%) |
| Other | 140 (33.3%) |
| Gastrointestinal | 105 (24.9%) |
| Constitutional | 20 (4.8%) |
| **Types of Grade ¾ AE’s Reported (N=422)** | |
| TRAE Only | 239 (56.6%) |
| TEAE Only | 90 (21.3%) |
| Both TRAE and TEAE’s | 48 (11.4%) |
| Not Specified | 45 (10.7%) |
| **Interval of Total AE Reporting (N=435)** | |
| Not Specified | 256 (58.9%) |
| Total Duration of Study | 143 (32.9%) |
| Cycle 1 and Total Duration | 13 (3.0%) |
| Not Reported | 13 (3.0%) |
| Cycle 1 | 5 (1.1%) |
| Other | 5 (1.1%) |
| **Most Common DLTs (N=426)** | |
| Other | 275 (64.6%) |
| Gastrointestinal | 72 (16.9%) |
| Hematologic | 54 (12.7%) |
| Constitutional | 25 (5.9%) |

Abbreviations: AE, adverse event; DLT, dose-limiting toxicity
